# Prof. Glyn Humphreys' Obituary

**DOI:** 10.3389/fncom.2016.00068

**Published:** 2016-07-06

**Authors:** Eirini Mavritsaki

**Affiliations:** Department of Psychology, Birmingham City UniversityBirmingham, UK

**Keywords:** Glyn Humphreys, visual perception and attention, neuropsychology, cognitive science, computational modeling

Professor Glyn Humphreys was born 28 December 1954 and passed away 14 January 2016.

Prof. Glyn Humphreys's early career was spent at the University of Bristol from where he was awarded his B.Sc. (1976) and Ph.D. (1980) in Psychology. At the early age of 24, Glyn was appointed to his first lectureship in the Department of Psychology in Birkbeck College, University of London. Almost all of his students were older than him! Through intellect and hard work, he made rapid academic progress, and was appointed as Professor of Psychology in 1988. While at Birkbeck, he was appointed the Ronald Tress Prize for advancements in his field, the first of many such awards. During his time in Birkbeck an equally important step in his life was made by meeting his wife Jane. Both of them moved to the University of Birmingham in 1989 where Glyn became the Head of the Department of Psychology, a position that kept for 15 years steering the department to the elite of institutes in the UK and worldwide. Most recently Glyn was the Watts Professor and Head of Experimental Psychology Department in Oxford University.

Glyn's contributions to research were enormous: he contributed to many areas including cognitive psychology, cognitive neuropsychology, computational modeling, social cognitive neuroscience, neuro-rehabilitation and cognitive diagnostics. Most notably his work on understanding the cognitive effects of stroke led to the development of the Birmingham Cognitive Screen in 2012. Glyn was also the president of Experimental Psychology Society from 2002 to 2004 and editor of three international journals. Glyn's work was recognized by numerous awards from the British Psychological Society (Spearman Medal, 1986, Cognitive Section Prize, 1999, President's award, 1999, Cognitive Section award, 2012), Humboldt Research, 1998, Royal Society (Wolfson merit award, 2007), University of Hong Kong (Distinguished Visiting Professor award, 2013) and European Society for Cognitive Psychology (Broadbent Prize for Cognitive Psychology, 2013). He was a fellow of the Royal Society of Medicine, the Humboldt Foundation, Ecoles des Hautes Etudes en Sciences Sociales and the British Academy. He was awarded an Honorary Life Fellowship of the Belgian Association for Psychological Science in 2002 and the British Psychological Society in 2012. Most recently, Glyn was awarded a Lifetime Achievement Award by the British Psychological Society in 2015.

Glyn was a giant in research and he collaborated with huge number of researchers from all over the world including Birkbeck, Birmingham, Oxford. His lab was notable for its “family feeling,” where new ideas were encouraged, fostered and supported. He was a mentor rather than supervisor, and his generous nature inspired those around him to aim higher, to think deeper, to be more responsible, to respect colleagues, to be compassionate and kind. Notably, the neuropsychological patients he worked with felt so respected and honored by him that they felt privileged to contribute to his research. In short Prof. Glyn Humphreys was a giant in science, humanity and kindness, and inspiration to many individuals around the globe.

Glyn survived by his wife Jane, his daughter Katie, his sons Iain and Alec and his grandchildren, Madeleine, Jake and Freddie.

## Author contributions

The author confirms being the sole contributor of this work and approved it for publication.

### Conflict of interest statement

The author declares that the research was conducted in the absence of any commercial or financial relationships that could be construed as a potential conflict of interest.

